# THE EFFECT OF AGE WHEN USING LLLT TO TREAT PAIN IN OLDER ADULTS

**DOI:** 10.1093/geroni/igac059.2537

**Published:** 2022-12-20

**Authors:** Alex Habegger, Danny Curtis, Dawn James, Alexis Kendrick, LaVona S Traywick, Antonio Varela, Teressa Brown, Reed Handlery

**Affiliations:** Arkansas College of Health Education, Fort Smith, Arkansas, United States; Arkansas Colleges of Health Education, Fort Smith, Arkansas, United States; Arkansas College of Health Education, Fort Smith, Arkansas, United States; Arkansas Colleges of Health Education, Fort Smith, Arkansas, United States; Arkansas Colleges of Health Education, Fort Smith, Arkansas, United States; Arkansas Colleges of Health Education, Fort Smith, Arkansas, United States; Arkansas Colleges of Health Education, Fort Smith, Arkansas, United States; Arkansas Colleges of Health Education, Fort Smith, Arkansas, United States

## Abstract

Low-level laser therapy (LLLT) is commonly used to treat pain; however, little is know about the effects of age on outcomes of the treatment. Participants included community dwelling adults (22 females, 9 males) with a mean age of 55 (n=31, range 29 to 77 years). Prior to participation, physical therapist examined each subject to determine appropriateness for treatment with LLLT. Following the examination, subjects received 12 sessions of LLLT using a Class 3B laser device. Intervention was administered by researchers trained in appropriate application of the intervention. The WALT guidelines and specific anatomical location of pain determined dosage of LLT. The most common site of pain or discomfort was hip and thigh pain (23%). Subjects completed the Patient-Specific functional Scale (PSFS) pre- and post-treatment and used the Numeric Pain Rating Scale (NPRS) before and after each intervention session. An analysis of the data showed that age was positively correlated (r=xx) and statistically significant (p < 0.05) with changes in both current and worst pain ratings and the first item on the PSFS. Age explained 13.4% of worst pain (p=0.024). Although not statistically significant, age explained 9.2% of the variability in current pain (p =0.054) and 3.9% of variability in patient identified changes in function (p = 0.147). Results suggest that older patients treated with LLLT may experience greater positive changes in pain than younger patients.

